# Supplementary material for: A Preliminary Assessment of the Nutraceutical Potential of Acai Berry (Euterpe sp.) as a Potential Natural Treatment for Alzheimer’s Disease
Source: Molecules. 2022 Jul 30;27(15):4891. doi: 10.3390/molecules27154891 (PMC9370152; doi:10.3390/molecules27154891)
Supplement: Supplementary file 1 [file molecules-27-04891-s001.zip › molecules-1828662-supplementary-revised.pdf]

Supplementary Table S1: Chemical compounds detected in acai berry extracts with possible cholinesterase inhibitor activity and recognized antioxidant activity. [99–172].

Compounds in Acai Extract	Cholinesterase Inhibitor Activity		Antioxidant Activity
	AChE	BuChE	
**Phenolic compounds and phenolic acids:**			
Ferulic acid	√	√	+ (DPPH, ABTS$^{•+}$)
Protocatechuic acid	√	√	+ (DPPH, ABTS$^{•+}$, FRAP)
Syringic acid	√	√	+ (DPPH, ABTS$^{•+}$)
Vanillic acid	√	√	+ (DPPH, ABTS$^{•+}$, FRAP)
Gallic acid	√	√	+ (DPPH, ABTS$^{•+}$, FRAP)
4-Hydroxybenzoic acid	√	√	+ (DPPPH, ABTS$^{•+}$)
Benzoic acid	√	√	+ (DPPH)
Coumaric acid	√	√	+ (DPPH, ABTS$^{•+}$)
3,4'-dihydroxy-3'-methoxypropiophenone	-	-	-
Protocatechuic acidmethyl ester	-	-	+ (DPPH)
3,4-Dihydroxybenzoic acid	-	-	+ (DPPH, ABTS$^{•+}$, FRAP)
2,5-Dihydroxybenzoic acid	-	-	+ (DPPH, ABTS$^{•+}$, FRAP)
Chlorogenic acid	√	√	+ (DPPH, ABTS$^{•+}$, FRAP)
Caffeic acid	√	√	+ (DPPH, ABTS$^{•+}$, $O^{2•-}$)
Trans-cinnamic acid	√	-	+ (DPPH, ABTS$^{•+}$)
Ellagic acid	√	√	+ (DPPH, ABTS$^{•+}$, FRAP)
3-hydroxy-1-(4-hydroxy-3,5-dimethoxyphenyl)-1-Propanone	-	-	-
**Lignans:**			
(+)-isolariciresinol	-	√	+ (DPPH)
(+)-5-methoxy-isolariciresinol	-	-	+ (DPPH)
Erythro-1-(4-hydroxy-3-methoxyphenyl)-2-[4-(3-Hydroxypropyl)-2-methoxyphenoxy]-1,3-propanediol	-	-	+ (SOD)
Threo-1-(4-hydroxy-3-methoxyphenyl)-2-[4-(3-hydroxypropyl)-2-methoxyphenoxy]-1,3-propanediol	-	-	+ (SOD)
(-)-(7R,8S)-dihydrodehydroconiferyl alcohol	-	-	-
(+)-(7R,8S)-5-methoxy-dihydrodehydroconiferyl alcohol	-	-	-
(+)-lariciresinol	√	√	+ (DPPH, ABTS$^{•+}$, $^{•}OH$, FRAP, SOD)
(+)-pinoresinol	√	-	+ (DPPH)
(+)-syringaresinol	√	-	+ (DPPH)
3-hydroxy-1-(4-hydroxy-3,5-dimethoxyphenyl)-1-propanone	-	-	+ (DPPH)
3,4'-dihydroxy-3'-methoxypropiophenone	-	-	-
Dihydroconiferyl alcohol	-	-	-
Protocatechuicacid methyl ester	-	-	+ (DPPH, ABTS$^{•+}$)
**Amino Acids:**			
Alanine	√	-	-
Lysine	√	-	+
Arginine	√	-	+
Methionine	ND	-	+
Aspartic acid	√	-	-
Phenylalanine	ND	-	-

Cysteine	√	-	+
Proline	√	-	-
Glutamic acid	ND	-	-
Serine	√	-	-
Glycine	-	-	-
Threonine	-	-	-
Histidine	ND	-	+
Tryptophan	ND	-	+
Hydroxyproline	-	-	+
Tyrosine	√	-	+
Isoleucine	-	-	-
Valine	√	-	-
Leucine	ND	-	-
**Anthocyanins:**			
Cyanidin 3-arabinoside	-	-	+ (ROS)
Cyanidin 3-arabionosylarabionoside	-	-	-
Cyanidin-3-O-glucoside	ND	-	+ (DPPH, ORAC)
Cyanidin-3-O-rutinoside	-	-	+ (H_2O_2, FRAP, $^{\bullet}NO$)
Pelargonidin-3-O-glucoside	-	-	+ (LPO, $O^{2\bullet-}$)
Peonidin-3-O-rutinoside	-	-	-
Cyanidin 3-sambubioside	-	-	-
Delphinidin 3-glucoside	-	-	+ (LPO)
Malvidin 3-glucoside (Oenin chloride)	-	-	+ (DPPH, ORAC)
Peonidin 3-glucoside	-	-	-
**Proanthocyanidins:**			
Catechin (+)	√	√	+ (DPPH, $ABTS^{\bullet+}$, FRAP)
Epicatechin (-)	√	√	+ (DPPH, $ABTS^{\bullet+}$)
Epigallocatechin	√	√	+ (DPPH, $ABTS^{\bullet+}$)
**Carotenoids:**			
α-carotene	-	-	+ (αTEAC, LPSC)
β-carotene	-	-	+ (DPPH, $ABTS^{\bullet+}$)
Lutein	√	-	+ (DPPH, $ABTS^{\bullet+}$, FRAP)
Zeaxanthin	-	-	+
**Flavonoids:**			
Apigenin	√	√	+ (DPPH, $ABTS^{\bullet+}$, ORAC)
Dihydrokaempferol	-	-	+ (DPPH, $ABTS^{\bullet+}$, FRAP)
Isovitexin	√	√	+ (DPPH, ORAC)
Luteolin	√	√	+ (DPPH, $ABTS^{\bullet+}$, FRAP, ORAC)
luteolin-C-8 -glucoside (orientin)	-	-	+ (DPPH, $ABTS^{\bullet+}$, FRAP)
luteolin-6-C-glucoside (homo-orientin)	-	-	+ (DPPH, $ABTS^{\bullet+}$, FRAP)
Quercetin	√	√	+ (DPPH, $ABTS^{\bullet+}$)
Scoparin	-	-	-
Taxifolin deoxyhexose or Taxifolin	-	-	+ (DPPH, $ABTS^{\bullet+}$)
Velutin	-	-	+ (ORAC)
Quercetin-3-O-rutinoside (rutin)	√	√	+ (DPPH, $ABTS^{\bullet+}$)
Vitexin	√	√	+ (DPPH, ORAC)
Kaempferol rutinoside	√	√	+ (DPPH, $ABTS^{\bullet+}$, ROS)
Kaempferol rhamnoside	-	-	+ (DPPH, $ABTS^{\bullet+}$, ROS)
Isoorientin	√	√	+ (DPPH, ORAC)
Crisoeirol	-	-	-
5,4'-dihydroxy-7, 3',5'-trimethoxyflavone	-	-	+ (ORAC)
Luteolin diglycoside	-	-	-
Procyanidin dimers	-	-	+ (DPPH, $ABTS^{\bullet+}$)

Chrysoeriol	-	-	+ (LPO, $O^{2\bullet-}$)
**Benzoquinone:**			
2,6-dimethoxy-1, 4-benzoquinone	√	√	+ ($O^{2\bullet-}$)
Monoterpenoids:			
(+)-menthiafolic acid	-	-	-
(E,Z)-2,6-dimethyl-2,6-octadiene-1,8-diol	-	-	-
(E,E)-2,6-dimethyl-2,6-octadiene-1,8-diol	-	-	-
**Norisoprenoids:**			
(-)-loliolide	√	√	+ (DPPH, $ONOO^-$)
(4R)-4-[(1E)-3-hydroxy-1-butenyl]-3,5,5-trimethyl-2-cyclohexen-1-one	-	-	-
Lipids sterols:			
ß-sitosterol	√	√	+ (DPPH, $ABTS^{\bullet+}$, H_2O_2, LPO)
Campesterol	-	-	+ (LPO)
Sitgmasterol	√	-	+ (LPO)
**Major Fatty Acids:**			
Monounsaturated Fatty Acids			
Oleic acid	-	-	+ (ROS)
Palmitoleic acid	-	-	-
Polyunsaturated Fatty Acids			
Linoleic acid	√	-	+ (DPPH, $ONOO^-$)
Linolenic acid	√	√	+ (DPPH, $ONOO^-$)
Saturated Fatty Acids			
Palmitic acid	√	√	-
Stearic acid	-	-	-
**Stilbenes:**			
Trans-resveratrol	√	√	+ (DPPH, $ABTS^{\bullet+}$, ORAC)
**Vitamins and minerals:**			
Calcium	√	-	-
Copper	√	-	-
Iron	√	-	-
Vitamin A	-	-	+ (DPPH, FRAP)
Vitamin C	-	-	+ (DPPH, $ABTS^{\bullet+}$, $O^{2\bullet-}$)

Abbreviation: αTEAC, α-tocopherol equivalent antioxidant capacity; ABTS, 2,2′-azino-bis(3-ethylbenzothiazoline-6-sulfonic; DPPH, 2,2-diphenyl-1-picrylhydrazyl; FRAP, ferric reducing antioxidant power; H_2O_2, hydrogen peroxide; $^{\bullet}OH$, hydroxyl radical scavenging assay; LPO, lipid peroxidation; LPSC, luminol-chemiluminescence peroxyl radical scavenging capacity; ND, not detected; $^{\bullet}NO$, nitric oxide; $O^{2\bullet-}$, superoxide anion radical; $ONOO^-$, peroxynitrite; ORAC, oxygen radical absorption capacity; ROS, reactive oxygen species; SOD, superoxide radical scavenging assay; (-), no data.
